# Prevalence and Antimicrobial Susceptibility of Indicator Organisms *Escherichia coli* and *Enterococcus* spp. Isolated from U.S. Animal Food, 2005–2011

**DOI:** 10.3390/microorganisms8071048

**Published:** 2020-07-15

**Authors:** Beilei Ge, Kelly J. Domesle, Stuart A. Gaines, Claudia Lam, Sonya M. Bodeis Jones, Qianru Yang, Sherry L. Ayers, Patrick F. McDermott

**Affiliations:** Division of Animal and Food Microbiology, Office of Research, Center for Veterinary Medicine, U.S. Food and Drug Administration, 8401 Muirkirk Road, Laurel, MD 20708, USA; kelly.domesle@fda.hhs.gov (K.J.D.); scsgaines@aol.com (S.A.G.); claudia.lam@fda.hhs.gov (C.L.); sonya.bodeis@fda.hhs.gov (S.M.B.J.); qianru.yang@fda.hhs.gov (Q.Y.); sherry.ayers@fda.hhs.gov (S.L.A.); patrick.mcdermott@fda.hhs.gov (P.F.M.)

**Keywords:** animal feed, animal food, antimicrobial susceptibility, *Enterococcus*, *Escherichia coli*, pet food, prevalence, resistance

## Abstract

The role animal food plays in the introduction of antimicrobial-resistant bacteria into the human food chain is not well understood. We conducted an analysis of 1025 samples (647 pet food and 378 animal feed) collected across the United States during 2005–2011 for two indicator organisms (*Escherichia coli* and *Enterococcus* spp.). The overall prevalence ranged from 12.5% for *E. coli* to 45.2% for *Enterococcus* spp., and 11.2% of samples harbored both organisms. Regardless of bacterial genus, animal feed had significantly higher prevalence than pet food (*p* < 0.001). A general downward trend in prevalence was observed from 2005 to 2009 followed by an upward trend thereafter. Among *E. coli* isolates (*n* = 241), resistance was highest to tetracycline (11.2%) and below 5% for fourteen other antimicrobials. Among *Enterococcus* spp. isolates (*n* = 1074), *Enterococcus faecium* (95.1%) was the predominant species. Resistance was most common to tetracycline (30.1%) and ciprofloxacin (10.7%), but below 10% for thirteen other antimicrobials. Multidrug-resistant organisms were observed among both *E. coli* and *Enterococcus* spp. isolates at 3.3%. Compared to National Antimicrobial Resistance Monitoring System (NARMS) 2011 retail meat and animal data, the overall resistance for both organisms was much lower in animal food. These findings help establish a historic baseline for the prevalence and antimicrobial resistance among U.S. animal food products and future efforts may be needed to monitor changes over time.

## 1. Introduction

Animal food is defined as “food for animals other than man and includes pet food, animal feed, and raw materials and ingredients” [1]. Most ingredients used to formulate animal feed rations (complete feeds) or pet food products are byproducts from the processing of plants (e.g., grains, oilseeds) and animals (e.g., rendered products, fish meal) that are fortified with mineral/vitamin supplements [2]. The United States, China, and Brazil are the top three animal-food-producing countries worldwide [3,4]. A recent survey from 145 countries showed that global animal food production remained steady in 2019, reaching a total of 1.126 billion metric tons [4]. Among 13 animal species, poultry (41.3%) had the largest share, followed by swine (23.2%), cattle (21.8%), aquaculture (3.6%), pets (2.5%), and equine (0.7%) [4]. As food-producing animals continue to make important contributions to our food supply, animal feed has become a critical component for producing safe food across the farm-to-table continuum [5]. Similarly, pet food plays an integral role in pet health and human health, with the percentages of U.S. households owning dogs and cats now at 38.4% and 25.4%, respectively [6]. Accordingly, global pet food production grew 4% in 2019 [4].

The World Health Organization (WHO) lists antimicrobial resistance (AMR) among the most urgent global health challenges [7]. In the U.S., more than 2.8 million drug-resistant infections occur annually, accounting for over 35,000 deaths [8]. One recent example highlighting the urgency of AMR is the global spread of a plasmid-mediated *mcr-1* gene conferring colistin resistance [9]. First reported in late 2015 in an *E. coli* strain from an intensive pig farm in China [10], this gene has subsequently been identified among diverse *Enterobacterales* species with a variety of plasmid types in dozens of countries [9]. In the U.S. and other countries, *mcr-1*-positve *E. coli* isolates have been found in food animals [11,12,13]. Considering that colistin is not an antimicrobial used in animal agriculture or veterinary medicine in the U.S., the source of colistin-resistant *E. coli* remains undetermined. Notably, a recent report indicated that imported food and feed were contributors to the introduction of *mcr-1*-positive *E. coli* to a low prevalence country [14].

To effectively combat AMR, a holistic One Health approach that integrates human, animal, and environmental sectors is imperative [15,16,17]. In October 2015, WHO launched the Global Antimicrobial Resistance Surveillance System (GLASS) to strengthen international AMR surveillance efforts [18]. Existing systems such as the U.S. National Antimicrobial Resistance Monitoring System (NARMS) [19], the Canadian Integrated Program for Antimicrobial Resistance Surveillance (CIPARS) [20], the Danish Integrated Antimicrobial Resistance Monitoring and Research Programme (DANMAP) [21], the Swedish Veterinary Antibiotic Resistance Monitoring Programme (SVARM) [22], and the European Union system for monitoring and collection of information on zoonoses [23] are all expanding to embrace the One Health concept.

Given that diverse ingredients of animal and plant origins are used to produce food for livestock animals and pets, animal food presents various opportunities for introducing antimicrobial-resistant bacteria and subsequently disseminating resistance genes via mobile genetic elements [24]. However, this commodity is seldomly included in AMR surveillance systems and available data are sparse [24]. For integrated surveillance of AMR in foodborne bacteria that applies a One Health approach, the WHO recommends including commensal, non-pathogenic, sentinel organisms such as generic *Escherichia coli* and *Enterococcus* spp. to provide information on the flow of Gram-negative and Gram-positive resistance traits in the food chain [25].

We conducted a pilot animal feed ingredient survey in 2002 and 2003, where *E. coli* and *Enterococcus* spp. were present in 39.3% and 86.6% of samples (*n* = 201), respectively, and resistance was highest to tetracycline in both *E. coli* (15.3%) and *Enterococcus* spp. (14.6%) [26]. Still, a more temporal and comprehensive approach is needed to better understand the potential impacts of animal food on AMR. Here we describe the findings on 1025 animal food samples collected between 2005 and 2011. The data suggest that *E. coli* and *Enterococcus* spp. were commonly present in U.S. animal food products with phenotypic resistance to some antimicrobials. None of the *E. coli* isolates possessed the *mcr-1* gene. Compared to NARMS 2011 retail meat and animal data, animal food overall had much lower resistance for both organisms.

## 2. Materials and Methods

### 2.1. Sample Collection

As part of the U.S. Food and Drug Administration (FDA)’s animal food surveillance programs, the Office of Research at the FDA’s Center for Veterinary Medicine (CVM) received investigational samples collected by the FDA’s Office of Regulatory Affairs (ORA) personnel across the United States [27,28]. The samples were analyzed for the presence and antimicrobial susceptibility of *E. coli* and *Enterococcus* spp. at the CVM.

From July 2005 to March 2011, a total of 1025 animal food samples (647 pet food and 378 animal feed) were randomly collected from representative animal food facilities across the United States. The types of samples collected included animal feed (for unspecified animal species), cattle feed, feed for minor species, horse feed, ingredients, medicated feed, poultry feed, swine feed, non-canned pet food/treats, and supplements for pets.

Specifically, the sample collection fell under the FDA’s Feed Contaminants Program [27] and the CVM Nationwide Pet Food Assignments [28]. The programs were established in 2002 and 2007, respectively, to monitor the trend of *Salmonella* contamination in animal food [29]. The collection, handling, and shipment of samples were in accordance with the FDA’s standard procedures [30]. Whenever the FDA ORA field investigators collected an official sample for *Salmonella* regulatory testing, they also collected an investigational sample (~ 300 g) and sent it to the CVM’s Office of Research for research purposes [27,28].

### 2.2. Bacterial Isolation and Identification

Upon arrival at the CVM laboratory, samples were stored at room temperature until processing. Each sample was aseptically divided into two subsamples. From each subsample, two 25 g portions were used for *E. coli* and *Enterococcus* isolation following methods described previously [26]. Media and reagents were obtained from BD Diagnostic Systems (Sparks, MD, USA) unless specified otherwise.

*E. coli* isolation followed the FDA’s *Bacteriological Analytical Manual* (BAM) Chapter 4 [31] with some modifications [26]. Briefly, 25 g of animal food was mixed with 225 mL of lauryl tryptose broth. After overnight incubation at 35 °C, an aliquot (100 µL) was transferred to 10 mL of *E. coli* (EC) broth and incubated at 45 °C for 24–48 h. At each interval, the EC broth showing gas formation was streaked onto eosin methylene blue agar and incubated at 35 °C overnight. Presumptive *E. coli* colonies (flat, dark green with metallic sheen) were subcultured on blood agar. *E. coli* isolates were confirmed by biochemical tests using the VITEK Legacy or VITEK 2 Compact system (bioMérieux, Marcy l’ Etoile, France).

For *Enterococcus* isolation, the NARMS method [19] was used with some modifications [26]. Briefly, 25 g of animal food was mixed with 225 mL of enterococcosel broth. After incubation at 45 °C for up to 48 h, the broth showing blackening was streaked onto enterococcosel agar and incubated at 35 °C overnight. Presumptive *Enterococcus* colonies (translucent with brownish-black to black zones) were subcultured on blood agar. Gram stain, catalase test, and PYR test were performed following the manufacturers’ instructions. *Enterococcus* species were determined using VITEK Legacy or VITEK 2 and PCR [32].

### 2.3. Antimicrobial Susceptibility Testing

Custom 96-well Sensititre panels designed by NARMS (TREK Diagnostic Systems, Oakwood Village, OH, USA) were used for antimicrobial susceptibility testing [19]. The minimal inhibitory concentrations (MICs) for all *E. coli* and *Enterococcus* isolates were determined by broth microdilution following guidelines of the Clinical and Laboratory Standards Institute (CLSI) [33]. Quality control organisms were *E. coli* ATCC 25922, *Enterococcus faecalis* ATCC 29212, *Pseudomonas aeruginosa* ATCC 27853, and *Staphylococcus aureus* ATCC 29213. The MIC data were interpreted according to CLSI guidelines [34] and NARMS breakpoints were used for ceftiofur and streptomycin in *E. coli* and kanamycin and tigecycline for *Enterococcus* spp. [35].

### 2.4. Screen for the mcr-1 Gene in E. coli

All *E. coli* isolates were screened for the presence of *mcr-1* retrospectively in 2016 by PCR with primers CLR5-F (5′-CGGTCAGTCCGTTTGTTC-3′) and CLR5-R (5′-CTTGGTCGGTCTGTAGGG-3′) [10]. A synthesized partial *mcr-1* gene (Integrated DNA Technologies, Coralville, IA, USA) was used as the positive control. The PCR mix (25 μL) consisted of 1× PCR buffer, 3 mM MgCl_2_, 0.2 mM each deoxynucleoside triphosphate (dNTP), 0.2 μM each primer (Integrated DNA Technologies, Coralville, IA, USA), 1.25 U of GoTaq Hot Start polymerase (Promega, Madison, WI, USA), and 2 µL of DNA template. The PCR program included initial denaturation at 94°C for 2 min followed by 25 cycles of denaturation at 94 °C for 30 s, primer annealing at 58 °C for 90 s, extension at 72 °C for 30 s, and a final extension at 72 °C for 10 min in a GeneAmp PCR system 9700 (Applied Biosystems, Foster City, CA, USA). PCR products (10 µL) were analyzed by gel electrophoresis and gel images were documented by the Gel Doc XR+ system (Bio-Rad, Hercules, CA, USA).

### 2.5. Statistical Analysis

Prevalence data sorted by target organism and animal food category or type were analyzed by using analysis of variance (R version 3.6.3 [36]). Trend analysis for prevalence was done using the Mann–Kendall trend test. Resistance data sorted by organism, antimicrobial agent, and animal food category were compared using the Chi-Square test. Prevalence and resistance data found in this study for animal feed were also compared with those from the NARMS 2011 report, which included testing for *E. coli* and *Enterococcus* spp. in retail meats (chicken, ground turkey, ground beef, and pork chops) and animals (chickens from hazard analysis and critical control point [HACCP] sampling only) [35]. Differences between the means were significant when *p* < 0.05.

## 3. Results

### 3.1. Prevalence of E. coli and Enterococcus spp.

Among 1025 animal food samples tested, 63.1% were non-canned pet food/treats and nutritional supplements for pets (*n* = 647) and 36.9% were animal feed including feed ingredients and complete feeds (*n* = 378). Animal byproducts (e.g., meat and bone meal, fish meal) comprised only a small portion (17.7%) of ingredient samples, and the majority (74.3%) were plant byproducts including canola meal, cottonseed meal, distillers grains, and soybean meal.

Overall, *E. coli* and *Enterococcus* spp. were recovered from 12.5% and 45.2% of samples, respectively, and 11.2% of samples harbored both organisms (Table 1). Regardless of bacterial genus, animal feed had significantly higher prevalence than pet food (*p* < 0.001). Within the animal feed category, the prevalence of *E. coli* was highest in cattle feed (45.9%) compared to other sources whereas the prevalence of *Enterococcus* spp. was significantly lower in ingredients (54%) compared to poultry feed and cattle feed (*p* < 0.05). Cattle feed also had the largest percentage of samples harboring both organisms (*p* < 0.05). Among the two pet food types, i.e., pet food/treats or supplements for pets, there was a significant difference observed in the prevalence of *Enterococcus* spp. (*p* < 0.01), but not *E. coli* (*p* > 0.05). A significantly smaller percentage of pet food (2.6%) contained both organisms compared to animal feed (25.9%) (*p* < 0.001) (Table 1).

**Figure 1:**
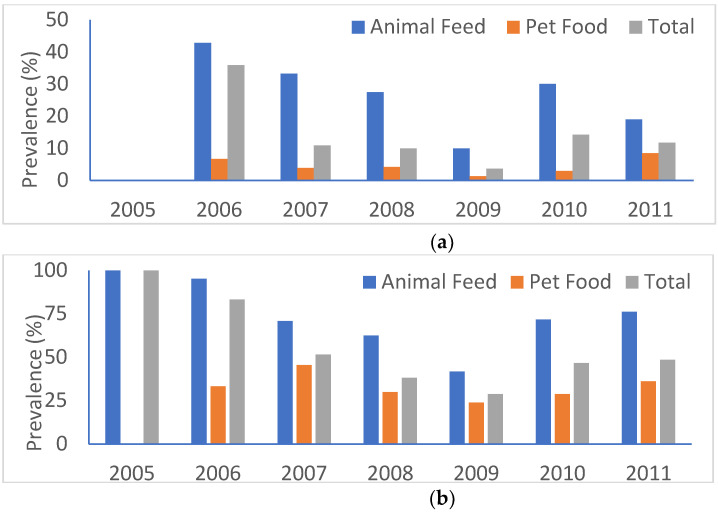
Prevalence of *E. coli* and *Enterococcus* spp. by year and animal food category. (**a**) *E. coli*; (**b**) *Enterococcus* spp.

Figure 1 shows the prevalence of *E. coli* and *Enterococcus* spp. by year and animal food category. Regardless of bacterial genus or animal food category, a general downward trend was observed up to 2009 followed by an upward trend thereafter (*p* < 0.05). The highest prevalence rate for *E. coli* (27/63, 42.9%) was in animal feed in 2006 whereas the two highest rates for *Enterococcus* spp. were in 2005 (4/4, 100%) and 2006 (60/63, 95.2%), both in animal feed. The lowest rates in animal feed for *E. coli* were 0% (0/4) in 2005 and 10% (6/60) in 2009 and for *Enterococcus* spp. 41.7% (25/60) in 2009. In pet food, the lowest rates were 1.3% (2/155) for *E. coli* and 23.9% (37/155) for *Enterococcus* spp., both in 2009 (Figure 1).

### 3.2. Composition of E. coli and Enterococcus Isolates

A total of 241 *E. coli* isolates were recovered, including 200 (83%) from animal feed and 41 (17%) from pet food (see isolate-level data in Supplementary Materials Table S1). 

Among 1074 *Enterococcus* isolates, 622 (57.9%) were recovered from animal feed and 452 (42.1%) from pet food. *Enterococcus faecium* (*n* = 1022; 95.1%) was the predominant species, followed by *Enterococcus hirae* (*n* = 12), *Enterococcus gallinarum* (*n* = 11), *Enterococcus casseliflavus* (*n* = 9), *Enterococcus durans* (*n* = 8), *Enterococcus faecalis* (*n* = 7), undetermined *Enterococcus* spp. (*n* = 3), and *Enterococcus mundtii* (*n* = 2). All nine *E. casseliflavus* isolates were recovered from animal feed whereas both *E. mundtii* isolates were from pet food (Table S1).

### 3.3. Absence of mcr-1 in E. coli

None of the *E. coli* isolates possessed the *mcr-1* gene by PCR, while the positive control consistently had good amplifications (data not shown).

### 3.4. Antimicrobial Susceptibility Profiles

All *E. coli* isolates (*n* = 241) were susceptible to 5 antimicrobial agents (amikacin, gentamicin, ceftiofur, ceftriaxone, and chloramphenicol) out of 15 antimicrobials on the NARMS Gram-negative panel (Table 2). Given the lack of resistance to two extended-spectrum β-lactams (ESBL, e.g., ceftiofur and ceftriaxone) on the panel, ESBL-producing *E. coli* was not found. Resistance was highest to tetracycline (11.2%) and below 5% for nine other agents. Isolates from pet food had significantly higher resistance rates than those from animal feed for amoxicillin-clavulanic acid and cefoxitin (*p* < 0.05) (Table 2). Both kanamycin-resistant *E. coli* isolates (one from cattle feed and one from pet food) were resistant to streptomycin, and both ciprofloxacin-resistant isolates (from a fish feed) were resistant to nalidixic acid (Table S1). 

All *Enterococcus* isolates (*n* = 1074) were tested using the NARMS Gram-positive panel. Two antimicrobial agents, flavomycin and lincomycin, were excluded from data analysis due to subsequent removal from the panel. Quinupristin/dalfopristin resistance was not reported for the seven *E. faecalis* isolates due to intrinsic resistance [37]. For the remaining 15 antimicrobials on the panel, all isolates were susceptible to 5 of them (gentamicin, vancomycin, tigecycline, nitrofurantoin, and linezolid) (Table 3). Although there was no vancomycin-resistant enterococci (VRE) found, two *E. faecium* isolates recovered from pet food exhibited intermediate susceptibility to vancomycin (MIC = 8 µg/mL) (Table S1). Resistance was common to tetracycline (30.1%) and ciprofloxacin (10.7%), but below 10% for eight other antimicrobials. For all ten antimicrobials, resistance rates did not differ significantly between isolates from animal feed and pet food (*p* > 0.05) except for quinupristin/dalfopristin (*p* < 0.05) (Table 3). All *Enterococcus* isolates demonstrating resistance to streptomycin, penicillin, chloramphenicol, and ciprofloxacin were *E. faecium* (Table S1). Thirteen isolates were resistant to daptomycin, including eight *E. faecium*, three *E. hirae*, one each *E. casseliflavus* and *E. gallinarum* with one *E. faecium* isolate (recovered from an animal feed sample for unspecified animal species) having an MIC of > 16 μg/mL (Table S1). All 41 tylosin-resistant *Enterococcus* isolates were also resistant to erythromycin (Table S1).

### 3.5. Multidrug Resistance

Among the 241 *E. coli* isolates, eight (3.3%) were multidrug-resistant, defined as resistant to three or more antimicrobial classes (Table 4). Two isolates were recovered from two separate pet food samples and six isolates from five different animal feed samples in three types, including animal feed (for unspecified animal species), cattle feed, and ingredient. Five isolates were recovered in 2010, two in 2006, and one in 2007 (Table 4).

Among the 1074 *Enterococcus* isolates, multidrug resistance was also observed in 35 (3.3%) of them, all being *E. faecium*, 17 (48.6%) of which were recovered from animal feed and 18 (51.4%) from pet food. Table 5 shows ten *E. faecium* isolates that were resistant to four or five antimicrobial classes. Five isolates were recovered from three different pet food samples and the other five isolates from five animal feed samples in four types, including animal feed (for unspecified animal species), feed for minor species, poultry feed, and swine feed. Four isolates were recovered in 2009, three in 2006, and one each in 2005, 2010, and 2011 (Table 5). An additional 25 *E. faecium* isolates were resistant to three antimicrobial classes (Table S1).

### 3.6. Comparison of animal feed data with NARMS 2011 Data

Prevalence rates for both *E. coli* and *Enterococcus* spp. in NARMS 2011 retail meats were significantly higher than those observed in animal feed (*p* < 0.001). Specifically, *E. coli* and *Enterococcus* prevalence was 55.7% and 87.2% in retail meats, respectively, while in animal feed, they were 28% and 70.4%, respectively. Prevalence data for NARMS 2011 animals were not available.

**Figure 2:**
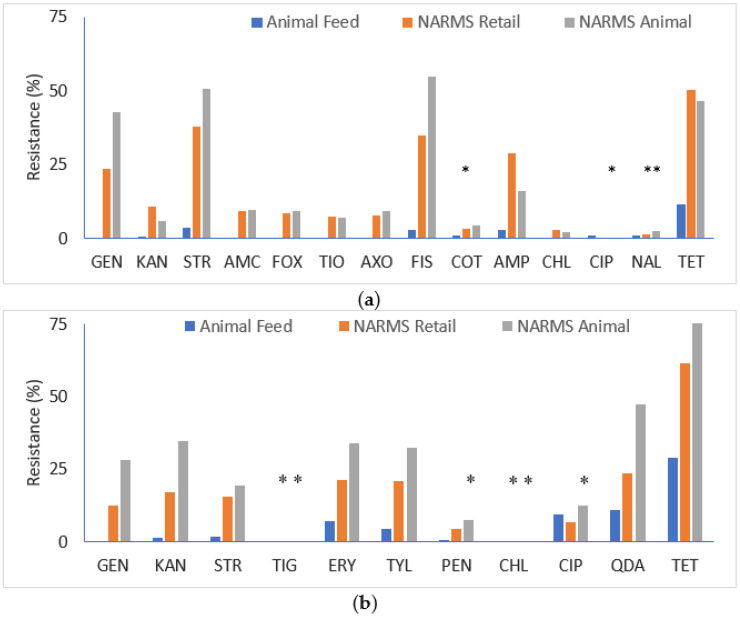
AMR of *E. coli* and *Enterococcus* spp. compared with NARMS 2011 retail meat and animal data. Each asterisk above the bar (*) indicates insignificant differences in resistance rate between isolates recovered from animal food and those from NARMS retail meat or NARMS animal. (**a**) *E. coli*; (**b**) *Enterococcus* spp. Abbreviations for antimicrobials are in (**a**): GEN, gentamicin; KAN, kanamycin; STR, streptomycin; AMC, amoxicillin-clavulanic acid; FOX, cefoxitin; TIO, ceftiofur; AXO, ceftriaxone; FIS, sulfisoxazole; COT, trimethoprim-sulfamethoxazole; AMP, ampicillin; CHL, chloramphenicol; CIP, ciprofloxacin; NAL, nalidixic acid; and TET, tetracycline; and in (**b**) GEN, gentamicin; KAN, kanamycin; STR, streptomycin; TIG, tigecycline; ERY, erythromycin; TYL, tylosin; PEN, penicillin; CHL, chloramphenicol; CIP, ciprofloxacin; QDA, quinupristin/dalfopristin; and TET, tetracycline. Antimicrobials not shown are amikacin for *E. coli*, and daptomycin, linezolid, nitrofurantoin, and vancomycin for *Enterococcus* spp. due to no resistance and/or unavailable NARMS 2011 data (amikacin and daptomycin).

When comparing resistance rates, *E. coli* and *Enterococcus* spp. isolated from animal feed had significantly lower rates than those recovered from either NARMS retail meats or animals (*p* < 0.05), with a few exceptions (Figure 2). For *E. coli,* there was no significant difference observed with ciprofloxacin (NARMS animal only), nalidixic acid (NARMS retail meat and animals), and trimethoprim-sulfamethoxazole (NARMS retail meat only) (*p* > 0.05), largely due to the low resistance prevalence to these antimicrobials in all sources. For *Enterococcus*, there was no significant difference observed for ciprofloxacin and penicillin (NARMS animal only), and chloramphenicol and tigecycline (NARMS retail meat and animals) (*p* > 0.05). The percentages of multidrug-resistant *E. coli* and *Enterococcus* spp. isolates were also significantly higher among NARMS 2011 isolates (*p* < 0.001).

## 4. Discussion

This longitudinal study is the largest to date examining the prevalence and antimicrobial susceptibility of two indicator organisms (*E. coli* and *Enterococcus* spp.) in animal food, providing a historic baseline for prevalence and resistance to antimicrobials with activity against Gram-negative and Gram-positive bacteria. Previous studies have largely focused on the status of pathogens (e.g., *Salmonella*, *Listeria monocytogenes*, and pathogenic *E. coli*) in livestock feed and pet food [24]. Pet food has been suggested as an important route of human exposure to AMR genes such as ESBL [38]. However, there has not been any survey of resistant bacteria other than *Salmonella* in dry pet food [29].

Samples analyzed in this study were convenience samples collected by the FDA’s Feed Contaminants Program [27] and the CVM Nationwide Pet Food Assignments [28], which were not random samples. Over 63% of samples were pet food, consisting of 564 non-canned pet foods/treats and 83 supplements for pets. Compared to animal feed, the prevalence of both *E. coli* (3.4% vs. 28%) and *Enterococcus* spp. (30.4% vs. 70.4%) was significantly lower in pet food (*p* < 0.001). This corroborates a previous study showing that aerobic plate counts were lowest in dog food (*ca.* 10^2^ CFU/g) and 1–2 logs higher in cattle feed and poultry feed [39]. The microbiological quality of starting ingredients, processing technologies, production environments, packaging methods, and storage and shipping conditions may have accounted for these differences.

Among the animal feed samples, cattle feed and poultry feed had the highest positive rates of both organisms (*E. coli*, 45.9% in cattle feed and 33.8% in poultry feed; *Enterococcus* spp., 85.2% in cattle feed and 86.2% in poultry feed). Ingredients comprised close to 30% of animal feed samples, with 29.2% positive for *E. coli* and 54% for *Enterococcus* spp. In our previous feed ingredient survey (201 samples), *E. coli* and *Enterococcus* spp. prevalence was 39.3% and 86.6%, respectively [26]. Samples in that survey were directly collected from processing plants in the United States while those in the present study had entered interstate commerce and/or were imported. These prevalence rates fell within the ranges reported in other earlier studies for *E. coli* in ingredients (32–43.5%), cattle feed (30.1–72.6%), and poultry feed (50%) [40,41,42] and for *Enterococcus* spp. in ingredients (66–81.3%) and poultry feed (100%) [42,43]. Interestingly, findings from this study and others [41,42] suggest a lower prevalence of *E. coli* and/or *Enterococcus* in feed ingredients compared to complete feeds. This may be partly explained by the high proportion of plant byproducts among our ingredient samples which tend to have much lower bacterial prevalence compared to animal byproducts [26]. The opposite (higher prevalence in ingredients compared to complete feed) was true for *Salmonella* as reported in the FDA’s animal food surveillance programs [29], suggesting the importance of pathogen-specific tests.

The downward trend observed from 2005/2006 to 2009 did coincide with *Salmonella* prevalence observed in the FDA’s surveillance programs [29]. From 2002 to 2009, the last year that data were made available, there was a significant reduction of *Salmonella* in animal food, most significant among ingredients and pet foods/treats (*p* < 0.05) [29]. In the present study, prevalence rates increased from 2009 to 2011 without known changes in sampling (types of samples etc.) or analysis, which may warrant recurrent surveillance efforts to monitor such trends. When comparing with NARMS 2011 retail meat data, prevalence rates for both organisms were significantly lower in animal food (*p* < 0.001). This is expected as *E. coli* and *Enterococcus* spp. are used as measures of fecal contamination which occur frequently during slaughtering of food animals. Interestingly, *E. faecium* predominated in animal food, whereas *E. faecalis* was much more prevalent among NARMS retail meat samples [35,44].

The potential development and dissemination of antimicrobial-resistant organisms and genes via animal food is an important public health concern [24]. A limited number of studies have examined the occurrence of antimicrobial-resistant *E. coli* and *Enterococcus* spp. in animal feed, mostly in feed ingredients [26,41,42,43,45,46], but none in pet food. For *E. coli*, resistance was most commonly identified for ampicillin, streptomycin, and tetracycline [26,42,45,46], as well as cephalothin [41,42], cefoxitin [41], and amoxicillin/clavulanic acid [41]. For instance, a study in Portugal [42] reported that among 58 feed ingredient isolates, resistance was common to tetracycline (27.6%), ampicillin (22.9%), streptomycin (19%), and cephalothin (15.2%), and to a lesser extent, trimethoprim/sulfamethoxazole (5.7%), chloramphenicol (4.8%), kanamycin (1%), and enrofloxacin (1%). In comparison, 105 *E. coli* isolates recovered from poultry feeds were frequently resistant to tetracycline (41.4%), ampicillin (22.4%), gentamicin (19%), and streptomycin (17%), while resistance to cephalothin, trimethoprim/sulfamethoxazole, kanamycin, apramycin, chloramphenicol, and enrofloxacin was <15% [42]. The *E. coli* resistance patterns observed in the present study agree with these previous findings [41,42], albeit at lower rates (<5% except for tetracycline which was 11.2%) and are comparable to our previous ingredient survey [26].

Fewer studies have examined the susceptibility/resistance of *Enterococcus* spp. isolates from animal food against a broad range of antimicrobials [26,42]. In the present study, *Enterococcus* spp. isolates demonstrated highest resistance to tetracycline (30.1%) and ciprofloxacin (10.7%), and below 10% to seven other antimicrobials; these rates were higher than those reported in our pilot ingredient survey [26], but lower than those observed in the Portuguese study [42], where authors reported resistance to rifampicin, erythromycin, nitrofurantoin, tetracycline, and ciprofloxacin at 59.8%, 21.6%, 21.2%. 18%, and 6.9% of 723 isolates, respectively. Further, among 414 isolates from poultry feeds, resistance to tetracycline (69.1%), rifampicin (58.5%), erythromycin (52.9%), and nitrofurantoin (36.2%) was frequently observed [42]. VRE isolates (mostly non-*E. faecalis* or non-*E. faecium*) were reported previously in feed ingredients [42,43] but not in the present study.

Mobile genetic elements such as plasmid-mediated colistin-resistant gene *mcr-1* play an important role in the transmission of AMR among humans, animals, and the environment, highlighting the importance of the One Health paradigm. A recent report indicated that imported food and feed were contributors to the introduction of *mcr-1*-positive *E. coli* to a low prevalence country [14]. We performed a retrospective analysis of all *E. coli* isolates for the presence of the *mcr-1* gene. Although not found in the present study, future surveys in animal food should consider including important AMR genes such as *mcr-1* for risk evaluation. Additionally, genetic characterization of other AMR genes and gene mutations to important antimicrobials such as ESBL and vancomycin should be performed, as the lack of phenotypic resistance may not definitely imply the absence of certain genes which may be expressed in different environmental or growth conditions.

Among existing AMR surveillance systems [19,20,21,22,23], indicator organisms in animal feed are not included. When comparing with NARMS 2011 retail meat and animal data, resistance rates in *E. coli* and *Enterococcus* spp. for most antimicrobials were significantly lower in animal food (*p* < 0.05). This may suggest that animal feed is not contributing (at any level of significance) to findings in retail products. Nonetheless, given the current scattered and sparse data on antimicrobial-resistant bacteria in animal feed, this may be a viable component for such programs using the One Health approach to better characterize and combat antimicrobial-resistant bacteria from humans, animals, and the environment.

## 5. Conclusions

This large survey demonstrated that indicator organisms *E. coli* and *Enterococcus* spp. were commonly present in animal feed and pet food products and there was an upward trend in prevalence from 2009–2011. Resistance to several antimicrobials was observed, albeit at a much lower rate than those observed in NARMS retail meat or animal sampling. Multidrug-resistant *E. coli* and *Enterococcus* spp. were observed in 3.3% of isolates. Because animal feed is at the very beginning of the farm-to-table continuum and pet food plays an integral role in animal and human health, these findings can serve as a historic baseline for understanding the prevalence and AMR in these commodities. However, it is important to note the samples in this study may not be representative of the current status of animal food due to regulatory policies implemented in the past decade (including the Food Safety Modernization Act regulation in animal food [47] and the Judicious Use of Medically Important Antimicrobial Drugs [48]) that could impact outcomes. Continued monitoring of animal feed as a component of One Health AMR surveillance may be warranted.

## Figures and Tables

**Table 1:** Prevalence of *E. coli* and *Enterococcus* spp. in 1025 animal food samples.

Animal Food Category/Type	No. of Samples	No. (%) of Positive Samples		
		*E. coli*	*Enterococcus*	Both
Animal Feed (all)	378	106 (28.0)^A^	266 (70.4)^A^	98 (25.9)^A^
Ingredients	113	33 (29.2)^ab^	61 (54.0)^b^	26 (23.0)^b^
Animal Feed (for unspecified animal species)	80	15 (18.8)^b^	52 (65.0)^ab^	14 (17.5)^b^
Poultry Feed	65	22 (33.8)^ab^	56 (86.2)^a^	22 (33.8)^ab^
Cattle Feed	61	28 (45.9)^a^	52 (85.2)^a^	28 (45.9)^a^
Feed for Minor Species	31	3 (9.7)^b^	22 (71.0)^ab^	3 (9.7)^b^
Swine Feed	12	3 (25.0)^ab^	10 (83.3)^ab^	3 (25.0)^ab^
Horse Feed	8	0 (0)^ab^	6 (75.0)^ab^	0 (0)^ab^
Medicated Feed	8	2 (25.0)^ab^	7 (87.5)^ab^	2 (25.0)^ab^
Pet Food (all)	647	22 (3.4)^B^	197 (30.4)^B^	17 (2.6)^B^
Pet Food/Treats	564	20 (3.5)^a^	182 (32.3)^a^	15 (2.7)^a^
Supplements for Pets	83	2 (2.4)^a^	15 (18.1)^b^	2 (2.4)^a^
Total	1025	128 (12.5)	463 (45.2)	115 (11.2)

In each column, percentages followed by different uppercase letters (A or B) were significantly different (*p* < 0.05) in prevalence between the two animal food categories (i.e., animal feed [all] and pet food [all]). In each column within animal feed or pet food categories, percentages followed by entirely different lowercase letters (a or b but not ab) were significantly different (*p* < 0.05) in prevalence within that animal food category.

**Table 2:** AMR (antimicrobial resistance) profiles among 241 *E. coli* isolates by animal food category.

Antimicrobial Class	Antimicrobial Agent	Resistant Breakpoint (μg/mL)	No. (%) of Resistant Isolates		
			All (*n* = 241)	Animal Feed (*n* = 200)	Pet Food (*n* = 41)
Aminoglycosides	Amikacin	≥ 64	0 (0)	0 (0)	0 (0)
	Gentamicin	≥ 16	0 (0)	0 (0)	0 (0)
	Kanamycin	≥ 64	2 (0.8)	1 (0.5)^A^	1 (2.4)^A^
	Streptomycin	≥ 64	11 (4.6)	7 (3.5)^A^	4 (9.8)^A^
Β-Lactam/β-Lactamase Inhibitor Combinations	Amoxicillin-Clavulanic Acid	≥ 32/16	1 (0.4)	0 (0)^B^	1 (2.4)^A^
Cephems	Cefoxitin	≥ 32	1 (0.4)	0 (0)^B^	1 (2.4)^A^
	Ceftiofur	≥ 8	0 (0)	0 (0)	0 (0)
	Ceftriaxone	≥ 4	0 (0)	0 (0)	0 (0)
Folate Pathway Inhibitors	Sulfisoxazole	≥ 512	7 (2.9)	6 (3)^A^	1 (2.4)^A^
	Trimethoprim-Sulfamethoxazole	≥ 4/76	2 (0.8)	2 (1)^A^	0 (0)^A^
Penicillins	Ampicillin	≥ 32	7 (2.9)	6 (3)^A^	1 (2.4)^A^
Phenicols	Chloramphenicol	≥ 32	0 (0)	0 (0)	0 (0)
Quinolones	Ciprofloxacin	≥ 1	2 (0.8)	2 (1)^A^	0 (0)^A^
	Nalidixic Acid	≥ 32	2 (0.8)	2 (1)^A^	0 (0)^A^
Tetracyclines	Tetracycline	≥ 16	27 (11.2)	23 (11.5)^A^	4 (9.8)^A^

NARMS breakpoints for ceftiofur and streptomycin were used. In each row, percentages followed by different uppercase letters (A or B) were significantly different (*p* < 0.05) in resistance rate between isolates recovered from animal feed and pet food.

**Table 3:** AMR profiles among 1074 *Enterococcus* isolates by animal food category.

Antimicrobial Class	Antimicrobial Agent	Resistant Breakpoint (μg/mL)	No. (%) of Resistant Isolates		
			All (*n* = 1074)	Animal Feed (*n* = 622)	Pet Food (*n* = 452)
Aminoglycosides	Gentamicin	> 500	0	0	0
	Kanamycin	≥ 1024	21 (2.0)	10 (1.6)^A^	11 (2.4)^A^
	Streptomycin	> 1000	16 (1.5)	11 (1.8)^A^	5 (1.1)^A^
Glycopeptides	Vancomycin	≥ 32	0	0	0
Glycylcyclines	Tigecycline	≥ 0.5	0	0	0
Lipopeptides	Daptomycin	≥ 8	13 (1.2)	6 (1.0)^A^	7 (1.5)^A^
Macrolides	Erythromycin	≥ 8	73 (6.8)	44 (7.1)^A^	29 (6.4)^A^
	Tylosin	≥ 32	41 (3.8)	29 (4.7)^A^	12 (2.7)^A^
Nitrofurans	Nitrofurantoin	≥ 128	0	0	0
Oxazolidinones	Linezolid	≥ 8	0	0	0
Penicillins	Penicillin	≥ 16	9 (0.8)	5 (0.8)^A^	4 (0.9)^A^
Phenicols	Chloramphenicol	≥ 32	2 (0.2)	2 (0.3)^A^	0^A^
Quinolones	Ciprofloxacin	≥ 4	115 (10.7)	60 (9.6)^A^	55 (12.2)^A^
Streptogramins	Quinupristin/Dalfopristin	≥ 4	101 (9.5)	69 (11.1)^A^	32 (7.1)^B^
Tetracyclines	Tetracycline	≥ 16	323 (30.1)	180 (28.9)^A^	143 (31.6)^A^

NARMS breakpoints kanamycin and tigecycline were used. Quinupristin/dalfopristin resistance was not reported for the seven *E. faecalis* isolates (3 recovered from animal feed and 4 from pet food) due to intrinsic resistance. In each row, percentages followed by different uppercase letters (A or B) were significantly different (*p* < 0.05) in resistance rate between isolates recovered from animal feed and pet food.

**Table 4:** Multidrug-resistant *E. coli* isolates (resistant to ≥ 3 antimicrobial classes).

No. of Antimicrobial Classes Resistant to	Resistance Pattern	Year	Animal Food Type
3	AMP-STR-TET	2006	Cattle feed
		2010	Ingredient
	FIS-STR-TET	2010	Ingredient
	FIS-[KAN-STR]-TET	2007	Pet food
		2010	Cattle feed
4	AMC-AMP-FOX-STR	2006	Pet food
	AMP-[COT-FIS]-STR-TET	2010	Animal feed (for unspecified animal species)
		2010	Animal feed (for unspecified animal species)

Antimicrobials in brackets belong to the same antimicrobial classes. Abbreviations for antimicrobials are: AMC, amoxicillin-clavulanic acid; AMP, ampicillin; COT, trimethoprim-sulfamethoxazole; FIS, sulfisoxazole; FOX, cefoxitin; KAN, kanamycin; STR, streptomycin; and TET, tetracycline.

**Table 5:** Multidrug-resistant *E. faecium* isolates that were resistant to 4 or 5 antimicrobial classes.

No. of Antimicrobial Classes Resistant to	Resistance Pattern	Year	Animal Food Type
4	CIP-PEN-QDA-TET	2006	Pet food
		2006	Pet food
	ERY-KAN-QDA-TET	2009	Pet food
	CIP-[ERY-TYL]-STR-TET	2006	Poultry feed
	[ERY-TYL]-[KAN-STR]-QDA-TET	2010	Feed for minor species
		2011	Animal feed (for unspecified animal species)
5	CIP-DAP-ERY-QDA-TET	2009	Pet food
		2009	Pet food
	CHL-[ERY-TYL]-QDA-STR-TET	2005	Swine feed
	CIP-[ERY-TYL]-[KAN-STR]-QDA-TET	2009	Feed for minor species

Antimicrobials in brackets belong to the same antimicrobial classes. Abbreviations for antimicrobials are: CHL, chloramphenicol; CIP, ciprofloxacin; DAP, daptomycin; ERY, erythromycin; KAN, kanamycin; PEN, penicillin; QDA, quinupristin/dalfopristin; STR, streptomycin; TET, tetracycline; and TYL, tylosin.

## Supplementary Materials

The following is available online at https://www.mdpi.com/2076-2607/8/7/1048/s1, Table S1: Isolate-level *E. coli* and *Enterococcus* spp. resistance data.
